# Combined MEK and Pi3′-kinase inhibition reveals synergy in targeting thyroid cancer *in vitro* and *in vivo*

**DOI:** 10.18632/oncotarget.15599

**Published:** 2017-02-21

**Authors:** Oussama ElMokh, Dorothée Ruffieux-Daidié, Matthias A Roelli, Amandine Stooss, Wayne A Phillips, Jürg Gertsch, Matthias S Dettmer, Roch-Philippe Charles

**Affiliations:** ^1^ Institut für Biochemie und Molekulare Medizin, Universität Bern, Bern, Switzerland; ^2^ Institut für Pathologie, Universität Bern, Bern, Switzerland; ^3^ Cancer Biology Laboratory, Peter MacCallum Cancer Centre, Melbourne, Australia

**Keywords:** genetically engineered mice, thyroid cancer, BRAF, Pi3K, combination treatment

## Abstract

Anaplastic thyroid cancers and radioiodine resistant thyroid cancer are posing a major treat since surgery combined with Iodine^131^ therapy is ineffective on them. Small-molecule inhibitors are presenting a new hope for patients, but often lead to drug resistance in many cancers. Based on the major mutations found in thyroid cancer, we propose the combination of a MEK inhibitor and a Pi3′-kinase inhibitor in pre-clinical models. We used human thyroid cancer cell lines and genetically engineered double mutant BRAF^V600E^ PIK3CA^H1047R^ mice to evaluate the effect of both inhibitors separately or in combination in terms of proliferation and signaling *in vitro*; tumor burden, histology, cell death induction and tumor markers expression *in vivo*. The combination of MEK and Pi’3-kinase inhibition shows a synergistic effect in term of proliferation and apoptosis induction through Survivin down-regulation *in vitro*. We show for the first time the effects of the combination of a MEK inhibitor and Pi3′-kinase inhibitor in a genetically engineered mouse model of aggressively lethal thyroid cancer. *In fine*, the two drugs cooperate to promote tumor shrinkage by inducing a proliferation arrest and an elevation of apoptosis *in vivo*. Moreover, a phenotypic reversion is also observed with a partial restoration of normal thyroid marker transcription, and thyroid cancer marker expression reduction.

In conclusion, combination therapy of MEK and Pi3′-kinase inhibition synergizes to target double mutant thyroid cancer *in vitro* and *in vivo*. This multidrug approach could readily be translated into clinical practice and bring new perspectives for the treatment of incurable thyroid carcinoma.

## INTRODUCTION

Thyroid cancer is the most common endocrine cancer. Its incidence has been rapidly increasing over the last decades but with a stable related morbidity rate [1]. Better detection of small tumors, thanks to the increased use of ultrasound and also histopathological over diagnosis, explains a part the rising incidence [2, 3]. The other part may be explained by increasing exposure to high levels of radiation including from radiation treatment to the head and neck, and/or fallout from nuclear power plant accidents or weapons testing [4].

About 62,500 new cases of thyroid cancer and more than 1,900 related deaths are estimated for 2015 in the United States according to the American Cancer Society, with a prevalence in women 3 times as high as in men [5]. Nevertheless, morbidity remains low compared to most other cancers since the more common subtypes of thyroid cancers have an excellent prognosis. Differentiated thyroid cancers including papillary (PTC) and follicular (FTC) thyroid cancers represent 90% of all thyroid cancers [6]. With 80% of cases, PTC is the most prevalent type. It responds very well to therapy consisting of surgery combined with therapeutic radioiodine (^131^I). However, patients with more advanced thyroid malignancies present the greatest challenge in the management of thyroid cancer. Anaplastic thyroid carcinoma (ATC) although rare, causes almost 50% of thyroid cancer related deaths [7]. This undifferentiated subtype is associated with a very poor prognosis and considered as one of the most aggressive cancers in humans with a 5-months median survival [8]. ATC is suspected to arise from well-differentiated thyroid carcinomas like PTC by further progression/dedifferentiation or to arise via poorly differentiated thyroid carcinomas (PDTC) [9, 10].

ATC presents as a diffuse invasion of structures in the neck such as trachea, esophagus, blood vessels and nerves, making surgery difficult to perform. Ultimately, this results in dyspnea and suffocation, which is the main cause of death in patients [11]. Nevertheless, metastasis is still observed in 10-20% of ATC cases, predominantly in the lung and bones [12].

BRAF is part of the Mitogen-activated protein kinase (MAPK) pathway leading to MAPK/ERK kinase (MEK) and Extracellular signal–regulated kinase (ERK) phosphorylation. It is commonly activated by somatic mutations in human cancer [13]. Detected in 8% of all cancers, BRAF mutations are common in melanoma and thyroid cancer with respective frequencies of 60% and 40%. The point mutation A1799T, coding for the V600E transversion, accounts for 90% of BRAF mutations. It is the most common mutation found in PTC (45% of patients), however, BRAF alone as a prognostic factor is controversial [14]. Data from sequencing of PDTC and ATC suggest, that the progression from well-differentiated thyroid cancers to ATC happens as a result of the cooperation between multiple acquired alterations such as mutations in *CTNNB1* (Human β-catenin gene), *PIK3CA* (Phosphatidylinositol 3-kinase catalytic subunit), *TP53* (Transformation related protein 53) and *PTEN* (Phosphatase and tensin homolog). Furthermore, copy number and epigenetic changes involving oncogenes have been described in this context [15].

Facing human cohort recruitment difficulties, animal models recapitulating the key genetic aspects of ATC are needed as a substitute. Currently, investigations are based on large retrospective cohort studies with a paucity of prospective randomized trials. Recently, significant efforts have been made in the direction of developing murine preclinical platforms allowing, on one hand, a better understanding of crucial events in the disease initiation and progression and, on the other hand, evaluation of rational targeted therapies. The role of the most common mutation BRAF^V600E^ in PTC initiation has been shown in several independent mouse models. First, thyroid-specific transgenic overexpression of BRAF^V600E^, induces goiter and invasive PTC with tall-cell features, which later transition to poorly differentiated carcinomas [16]. Later, a mouse model using thyroid-specific recombination to mimick the point mutation event observed in patients, demonstrated that BRAF^V600E^ expression was sufficient to drive PTC formation [17].

Furthermore, several mouse models associating BRAF mutations to other oncogenic mutations have been described [18]. Deletion or dominant negative mutation of TP53 leads to progression from PTC to ATC [19]. Another model using PIK3CA^H1047R^ and BRAF^V600E^ expression in the thyroid showed that the combination cooperates to promote tumor progression to ATC, characterized by local invasion and subsequent death of the mice by suffocation [20], resembling very closely the human disease.

Thyroid tumors carrying BRAF^V600E^ mutations are assumed to be exquisitely dependent on the oncoprotein activity for viability. Thus, pharmacological inhibition is associated with tumor regression and a partial restoration of the differentiated phenotype [17]. However, mono-therapies targeting one component of the MAPK pathway in human patients are only transiently effective in some cancers such as melanoma, since resistance arises frequently after few months [21]. This resistance is due to reactivation of the ERK signaling pathway via various mechanisms [22, 23].

We hypothesize that targeting both MAPK and Phosphoinositide 3′-kinase (Pi3′-kinase) pathways by inhibiting the mutated enzymes that drive the development of the disease or their downstream targets MEK would have clinically measurable beneficial effect. To address this question, we started by studying the effect of drugs targeting the two pathways in human ATC cell lines. We looked at eventual cooperation and possible molecular mechanisms driving it. We used a mouse model of aggressive PTC that progresses to ATC (thyrocyte-specific expression of BRAF^V600E^ and PIK3CA^H1047R^ [20]) to evaluate the combination effects of MEK and PI3′-kinase inhibition in a pre-clinical context. The tumor burden measured by ultrasound was used as a read-out, along with the evaluation of the expression of clinical markers of thyroid cancer and the transcription of normal thyroid markers in tumor tissue from treated mice.

## RESULTS

### MEK inhibition synergizes with Pi′3-kinase inhibition in ATC cell lines

Since cancer is driven by multiple mutational events, multidrug approaches are now becoming standard to tackle tumors. Moreover, most mono-therapies are showing mitigated results and emergence of resistance mechanisms with time. We wanted to assess if targeting MEK downstream of BRAF in combination with a Pi3′-kinase inhibitor induces stronger proliferation reduction than any drug alone in human ATC cell lines. Three cell lines with BRAF^V600E^ mutations were selected. The SW1736 cell line has no other mutations reported. However, OCUT-2 cells have an additional PIK3CA^H1047R^ mutation and 8505c cells have both *TP53* and *CDKN2A* (Cyclin Dependent Kinase Inhibitor 2) deletions.

We used PD-325901, which is a potent and selective MEK1/2 inhibitor that has reached phase II clinical trials for advanced non-small lung cancer, as a single agent [24]. For Pi3′-kinase inhibition, we used GDC-0941, currently studied in phase Ib/II clinical trials for solid tumors [25].

**Figure 1:**
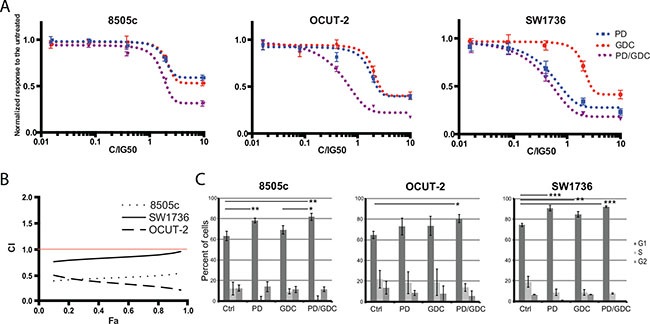
Drug combination synergistically inhibits thyroid cancer cell proliferation (**A**) Differential response to single and combination treatment with MEK and PI3′-kinase inhibitors: Human anaplastic thyroid cancer cells were treated with increasing concentrations of single drugs PD-325901, GDC-0941 or the combination at a constant ratio for about 3 doubling population times. All drug concentrations were normalized to IG50 equivalents of single agents (see Materials and Methods). Optical Density values were normalized to the untreated. All the measurements were done in triplicate and the mean was represented with SEM. The constant ratio approach allows figuring out obvious synergistic effect between drugs and calculating the combination index. (**B**) Combination index (CI) as a function of Fraction affected (Fa): Combination index curves were calculated using Compusyn Software 2.0 based on Chou and Talalay method. The horizontal red line indicates the synergistic effect threshold. (**C**) Cell cycle analysis on cells treated with single drug or the combination: Cell cycle analysis was performed on 8505c, OCUT-2 and SW1736 treated for 24 h with PD-325901 at 100 nM and/or GDC-0941 at 1 μM. Bars represent percentage of cells in each cell cycle stage as the mean of triplicates from independent experiments with the calculated Standard Deviation.

To test the nature of the collaboration between the two drugs, we performed a synergy test by incubating cells with a range of concentrations from 0.016 fold of IG_50_ to 10 fold of IG_50_ obtained by 5-fold serial dilutions. The specific Half-maximal inhibitory growth concentrations (IG_50_) of PD-325901 and GDC-0941 used to calculate synergy, and previously determined for each cell line experimentally, were respectively around 10 nM and 200 nM in both OCUT-2 and SW1736 cell lines. In 8505c cell line, the IG_50_ concentrations were 20 nM and 300 nM. In 8505c and OCUT-2 cells, a greater inhibition of proliferation was obtained with the combination, as seen by the left shift of the combination curve compared to the single treatment curves. This effect was less pronounced in SW1736 cells (Figure 1A). The constant concentration ratio approach (See Material and Methods) allowed us to calculate the combination index from the obtained proliferation data based on the method of Chou and Talalay [26].

The strongest synergistic effect was obtained in OCUT-2 cell lines followed by 8505c. In SW1736 The combination index was ranged from 0.8 to 0.9 over the concentrations tested. Even though 1.0 is the mathematical threshold, 0.85 is considered a more significant threshold since, statistically, the combination index cannot be considered to show any effect deviating from the additivity between 0.85 and 1.0 [27]. We can say then that in this cell line we have, in the range of concentrations used, an effect between additive and moderately synergistic (Figure 1B).

**Figure 2:**
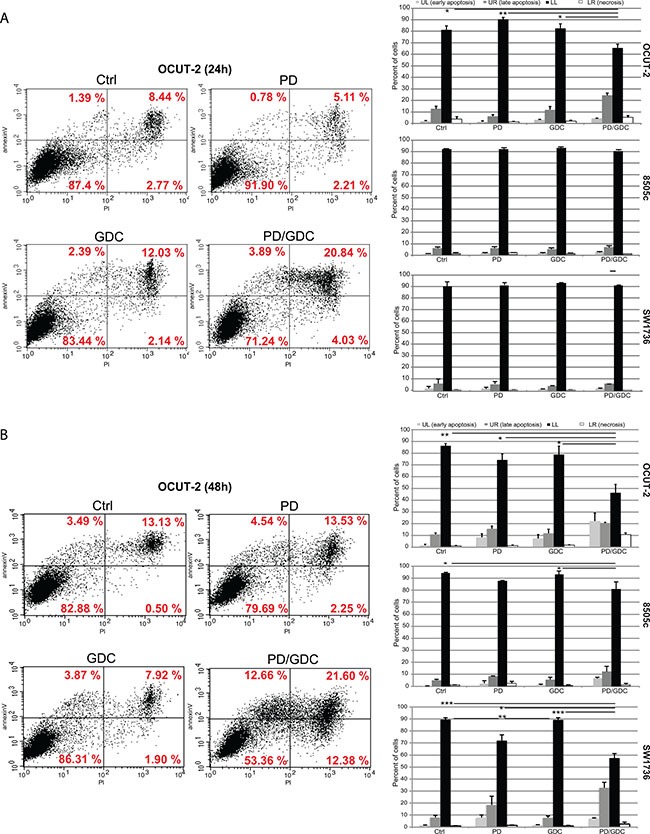
Drug combination synergistically induces apoptosis in anaplastic thyroid cancer cell lines (**A**) Flow cytometry analysis of OCUT-2 cells treated with 100 nM of PD-325901, 1 μM of GDC-0941 or the combination for 24 h stained with FITC annexinV and PI. Apoptosis was detected by FITC annexinV binding to the cells. Quadrant Lower Left, FITC annexinV(−) PI(−) represents living cells. Quadrant Lower Right, PI(+) represents cells undergoing necrosis. Quadrant Upper Right, FITC annexinV(+) PI(+) represents cells in the late apoptosis and undergoing secondary necrosis. Quadrant Upper Left, FITC annexinV (+) PI(−) are cells in early apoptosis. The histograms represent the quantifications of intact cells, early apoptotic cells, late apoptotic cells and necrotic cells in the 3 cell lines after 24 h treatments as the mean of triplicate from 3 independent experiments. Error bars represent SEM. (**B**) Flow cytometry analysis of OCUT-2 cells treated with 100 nM of PD-325901, 1 μM of GDC-0941 or the combination for 48 h stained with FITC annexinV and PI. The histograms represent the quantifications of intact cells, early apoptotic cells, late apoptotic cells and necrotic cells in the 3 cell lines after 48 h treatments as the mean of triplicate from 3 independent experiments. Error bars represent SEM.

To understand the mechanism underlying the inhibition, we investigated the effect of the drugs on cell cycling. PD-325901 alone or in combination with GDC-0941 induced a G1 cycle arrest in SW1736 and 8505c cell lines. However, in OCUT-2, a significant effect was only observed for the combination (Figure 1C). Altogether this moderate additional cytostatic effect of the drug combination could not explain the synergy in terms of reduction in cell number. Therefore, we hypothesized that induction of cell death could explain the difference observed. We then performed an apoptosis assay by flow cytometry using annexinV and Propidium Iodide (PI). Only the OCUT-2 cell line already showed increased apoptosis (double positive annexinV and PI cells) when treated with the combination for 24 h (Figure 2A). However, after 48 h of combination treatment, all three cell lines (Figure 2B and Supplementary Figure 1) had elevated double positive annexinV/PI cells (late apoptosis) and annexinV positive cells (early apoptosis). The most remarkable observation was the significant decrease of intact cells with drug combinations in all three cell lines after 48 h.

**Figure 3:**
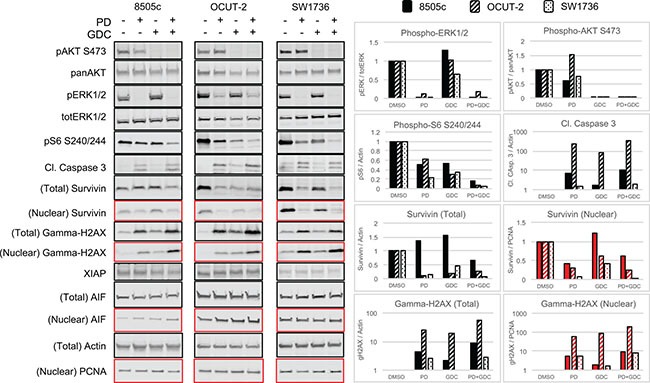
The combination PD-325901/GDC-0941 inhibits MAPK and Pi3′-kinase pathways and leads to caspase dependent cell death enhanced by Survivin downregulation Western blot showing the expression of component of signaling pathways and proteins involved in apoptosis caspase dependent and caspase independent in the 3 ATC cell lines treated for 24 h with single treatments or the combination PD-325901/GDC-0941. The quadrants in red correspond to the nuclear fraction and in black to the total extract.

To further investigate the observed apoptosis, we performed western blot analysis. ERK1/2 and AKT phosphorylation were assessed first to demonstrate the drug efficiency. ERK1/2 phosphorylation ratio (p-ERK1/2 normalized to total ERK) was strongly decreased in all cell lines when treated with PD-325901 alone or in combination with GDC-0941. Similarly, GDC-0941 induced a strong reduction of AKT phosphorylation ratio (Figure 3). Ribosomal S6 phosphorylation was affected by both treatments and the combination resulted in further de-phosphorylation of S6 showing a collaborative effect of the two drugs at the level of this signaling node.

Cell death was also investigated by detection of cleaved caspase 3 (CC3). In all three cell lines, CC3 was mainly driven by PD-325901 treatment, but still showed a further elevation upon addition of GDC-0941. We additionally tested modulators of caspase activity like Survivin: there we could detect a down regulation of Survivin in whole protein lysates that was also found in the drug combination. This conjunction of CC3 elevation combined with down regulation of Survivin could very likely explain the increased cell death that was observed with a time shift of 24 to 48 h. Apoptotic cell death was assessed here by monitoring DNA fragmentation with gamma-H2AX that was found increased under drug treatments in both whole cell lysates and nuclear extracts (Figure 3) in the three cell lines. We also tested alternative mechanisms of apoptosis like X-chromosome-linked inhibitor of apoptosis protein (XIAP) down regulation or induction of the apoptosis inducing factor (AIF) but these did not show any differences in expression (Figure 3).

### BRAF^V600E^ and PIK3CA^H1047R^ tumors regressed under MEK inhibitor treatment and further if combined with Pi3′-kinase inhibitor

**Figure 4:**
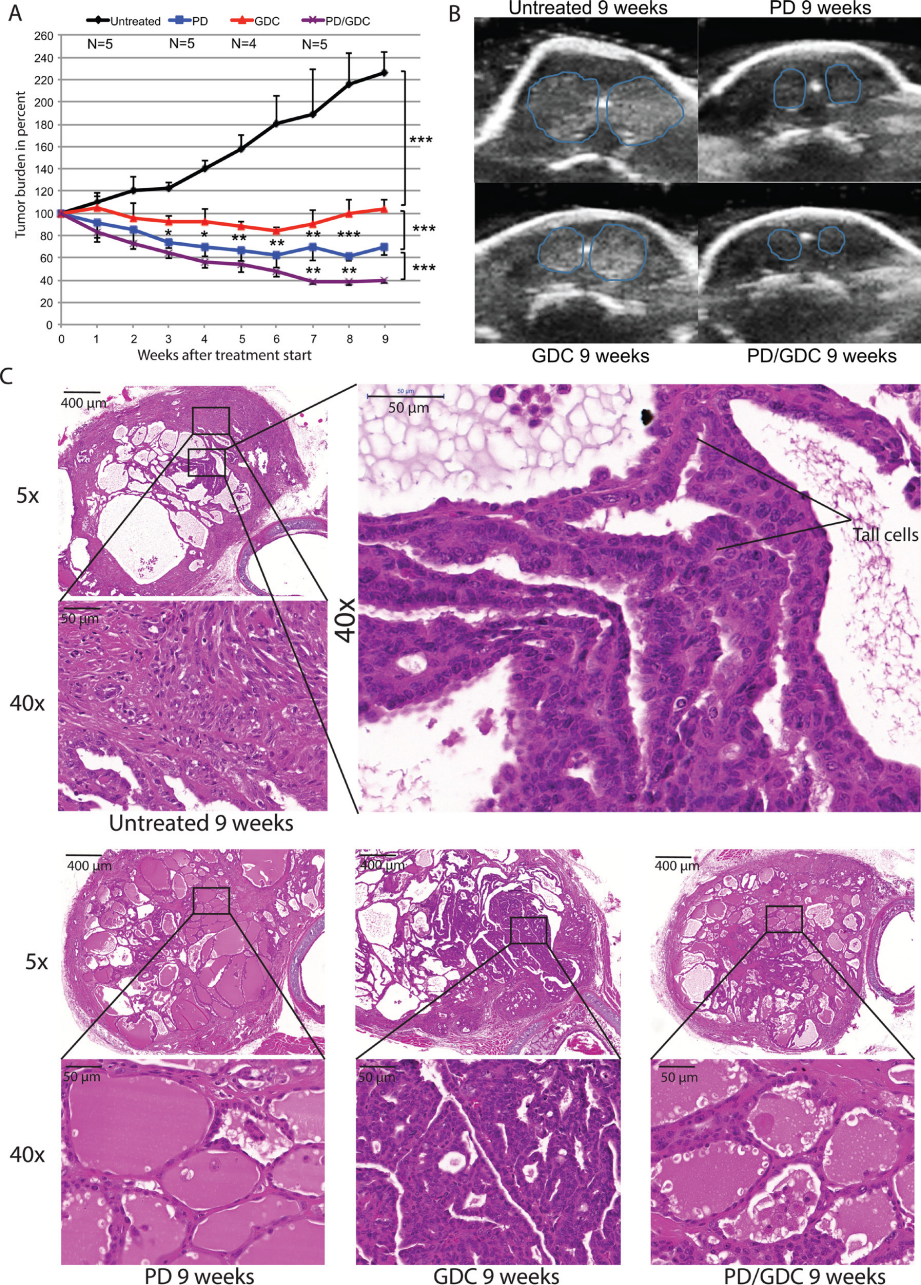
The combination is more beneficial after 9 weeks of treatment in terms of tumor burden reduction compared to PD alone and shows histology improvement (**A**) Thyroid tumor burden during treatment measured by ultrasound imaging expressed in percentage of the starting tumor burden. The mean of each treated group was calculated with SEM. 2-way ANOVA test with Tukey as post hoc test were used for multiple comparison (**B**) Representative pictures of ultrasound pictures at the end of the treatment (9 weeks). (**C**) Hematoxylin and Eosin stainings at 5× and 40× magnification of representative thyroid tissues after 9 weeks of treatments of mice by oral gavage with Vehicle, PD-325901 at 5 mg/kg, GDC-0941 at 50 mg/kg and combination.

To test if PD-325901 and GDC-0941 would also cooperate *in vivo*, we used a previously described mouse model of thyroid cancer [20]. In this model, the endogenous expression of BRAF^V600E^ and PIK3CA^H1047R^ can be induced by tamoxifen specifically in thyrocytes at 30 days of age. After tamoxifen injection, mice rapidly develop PTC that progresses to ATC and death of the mouse within 3 to 6 months after the drug-induced mutational induction [20]. Treatment of mice with drugs was started 2 months after tumor induction and ultrasound measurements were performed weekly to assess the tumor burden (Figure 4A–4B). Untreated mice display a steadily increasing tumor burden reaching 220% over the time of the experiment. GDC-0941 treated mice presented a 20% tumor burden reduction at 6 weeks of treatment but regained their starting size after 9 weeks. Interestingly, PD-325901 treated mice presented a 40% tumor burden reduction after 6 weeks of treatment and remained stable for the rest of the experiment. Strikingly, the combination treated animals had a more pronounced response with a 60% tumor burden reduction after 7 weeks (Figure 4A).

After 9 weeks of treatment, we seemed to have achieved a maximum stable response for the combo-treated animals. Therefore, three mice from each group were dissected and thyroids were processed for histological analysis. Untreated mice showed an expected histology with PTC areas, tall cell morphology and phenotypic progression to ATC in some areas. Interestingly, PD-325901 treated mice showed a clear improvement in histology with some almost normal follicles and smaller PTC areas. GDC-0941 did not induce a beneficial effect at the histological level. Finally, mice treated with the combination, although resulting in smaller sections, seemed to have a similar histological presentation to PD-325901 alone treated animals (Figure 4C).

**Figure 5:**
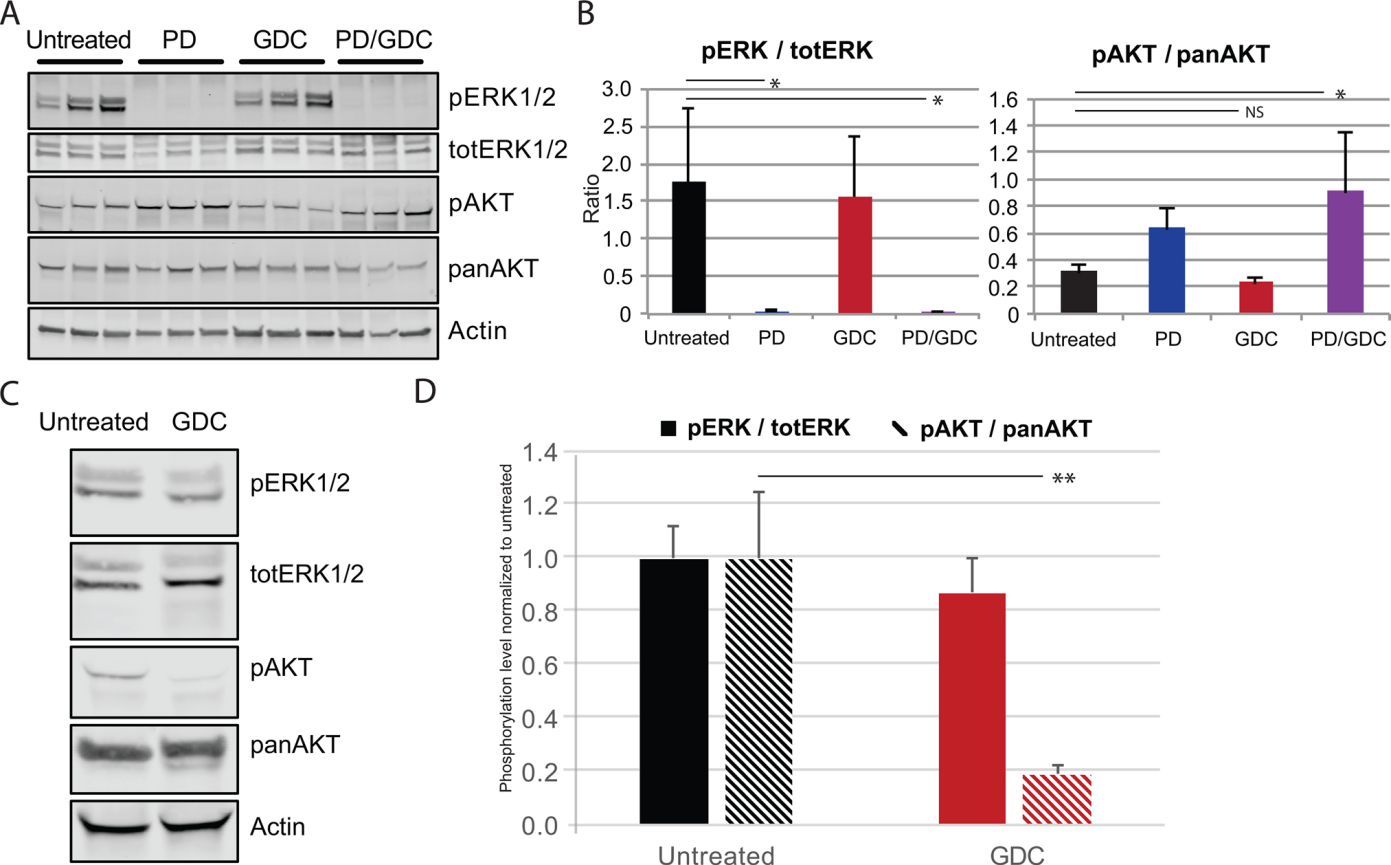
PD and GDC drugs are hitting their target *in vivo* based on the decreased phosphorylation of ERK and AKT (**A**) Western blot showing the expression of pERK1/2, pAKT, pERK, panAKT in the tumors of control and treated mice for 9 weeks (**B**) Quantification of ratio pERK/totERK and pAKT/panAKT after 9 weeks of treatment. Means are represented with SEM and One-way Anova test with Dunnett as post hoc were used to assess statistical significance (**C**) Western blot showing the expression of pERK1/2, pAKT, pERK, panAKT in the tumor of control and treated mice with 1 shot GDC-0941 at 50 mg/kg by oral gavage. (**D**) Quantification of ratio pERK/totERK and pAKT/panAKT after short treatment with GDC-0941 (1× 50 mg/kg). the statistical significance was evaluated using a *T* test.

In terms of signaling, ERK and AKT phosphorylation ratios were assessed by western blot after 9 weeks of treatment to demonstrate the efficacy of the drug regimen. PD-325901 alone or in combination was able to induce a strong dephosphorylation of ERK1/2. However, GDC-0941 induced only a small non-significant decrease in terms of AKT phosphorylation. In tumors from PD-325901 treated animals there was a tendency to elevated AKT phosphorylation, that became significant in the combination treatment (Figure 5A–5B). The lack of effect of GDC-0941 on AKT-phosphorylation was unexpected. Therefore, we tested the drug on mice over a shorter period of time. Another group of mice was treated with only one dose of GDC-0941 by oral gavage then sacrificed 4 hours later. In this setting, western blots showed a much stronger reduction of AKT phosphorylation (-80%) (Figure 5C–5D) showing that the drug was efficiently inhibiting Pi3′-kinase, even if the effect on the read-out pAKT does not seem to last under a chronic treatment.

### Drug removal induces immediate regrowth of all tumors

**Figure 6:**
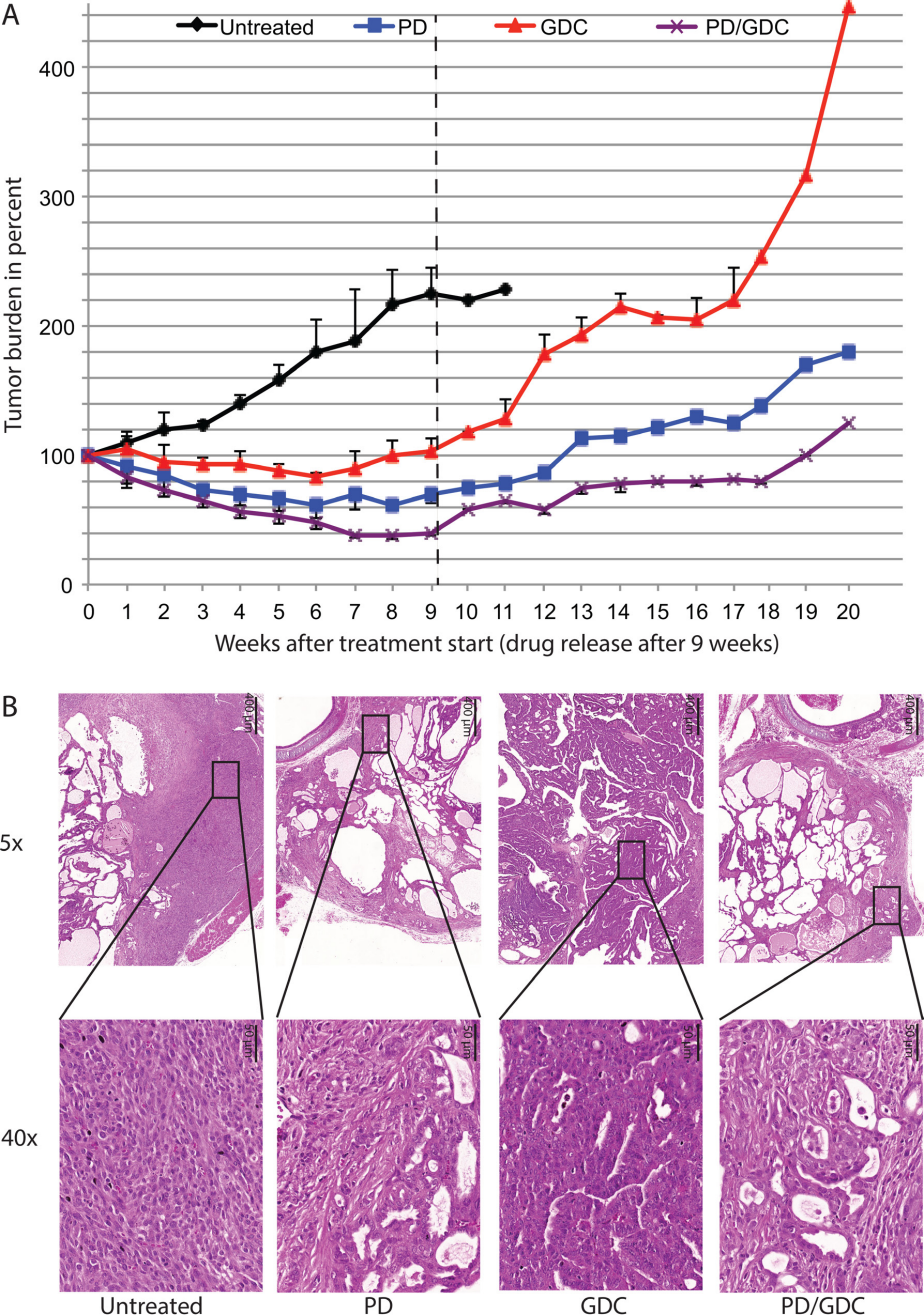
Tumors regrow after treatment release with different rate and seem to lose the histological improvement observed under treatment (**A**) Tumor burden during treatment (9 weeks) and regrowth after treatment removal up to 20 weeks measured on remaining mice. (**B**) Hematoxylin and Eosin staining at 5× and 40× magnification of representative thyroids tissues after treatment removal with a control mouse euthanized at week 11 after reaching endpoint. PD-325901, GDC-0941 and combination treated mice were sacrificed at week 20 (11 weeks after drug release).

To test if the treatment was curative we performed tumor burden measurement after drug release in two mice remaining from the PD-325901 and GDC-0941 treatment groups and one mouse remaining from the combo-treated group (after week 9). From the ultrasound data acquired in this configuration we could clearly conclude that in all treated mice living tumor cells were remaining and capable of re-growing suggesting that the treatments were not curative (Figure 6A).

Looking at the histology of re-growth, untreated mice displayed features of progression with large areas of ATC. GDC-0941 treated mice although presenting the biggest tumors, look mostly like regular PTC and did not seem to have progressed to ATC. Thyroids in mice treated with PD-325901 or the combination no longer showed the histological improvement seen after treatment and presented with an aggressive phenotype (Figure 6B).

### BRAF^V600E^ and PIK3CA^H1047R^ tumors regressed under MEK and Pi3′-kinase inhibition by reducing cell proliferation and increasing cell death

**Figure 7:**
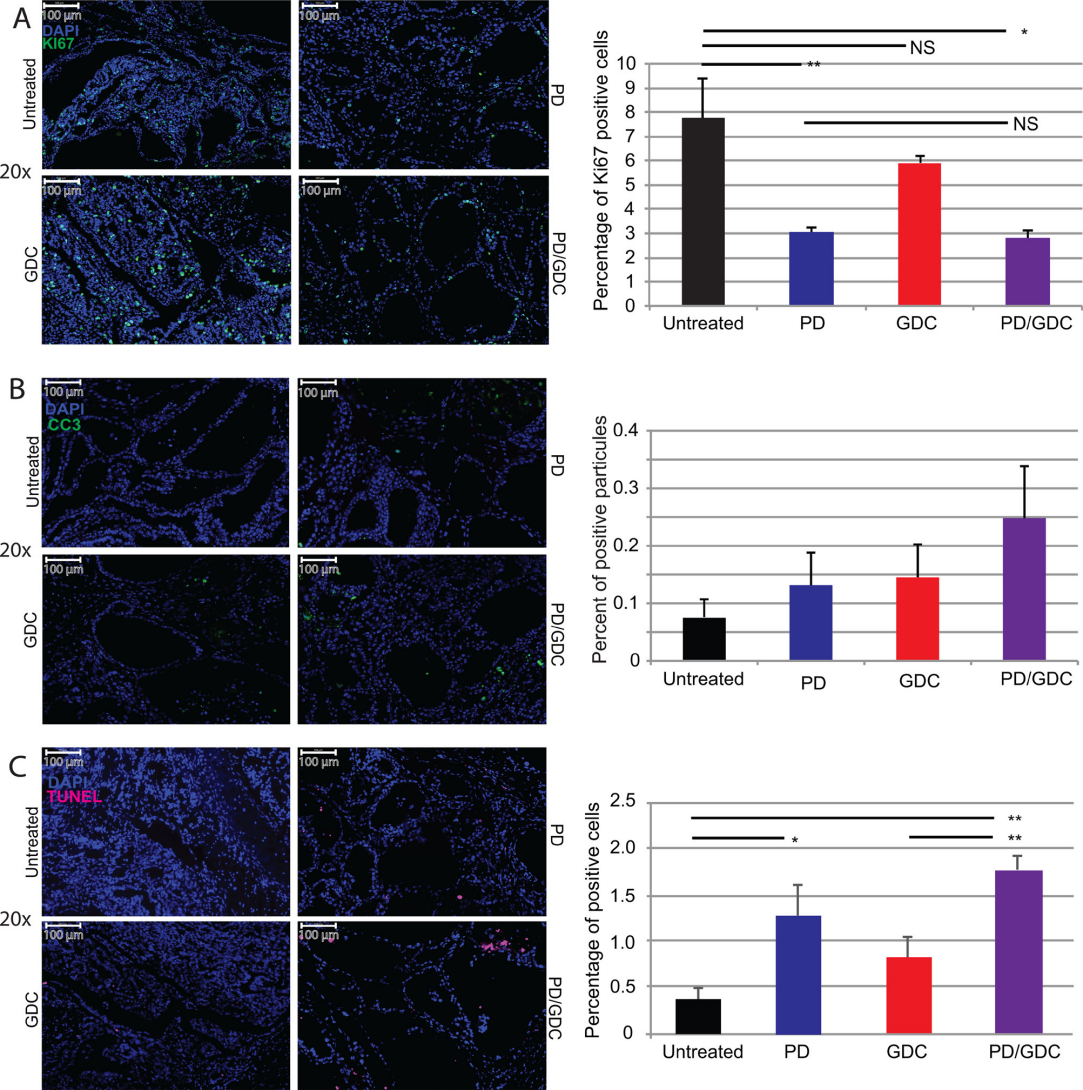
Drug treatments induce a decrease in proliferation and increased cell death Representative immunofluorescence images of isolated thyroid tissues and corresponding quantification. The tissues were isolated after 9 weeks of treatment. (**A**) Blue: DAPI and Green: Ki67. The graph shows the percentage of Ki67 positive cells. (B) Green: CC3. The graph displays the count of CC3 apoptotic bodies normalized to nuclei number. (**C**) Green: TUNEL. The graph depicts the count of TUNEL positive apoptotic bodies normalized to the number of nuclei. All quantifications were performed on whole tumor sections using Quant Center software from 3DHISTECH. The mean of different measurements (thyroid lobes) are represented for each treatment group with SEM. One-way ANOVA test with Tukey as post hoc test were used to calculate statistical significance.

In order to understand the mechanisms driving the tumor regression, we performed immunofluorescence staining for Ki67, CC3 and DNA fragmentation (Terminal deoxynucleotidyl transferase dUTP nick end labeling or TUNEL) on thyroid sections of the 9 weeks treated mice. The proliferation index was significantly reduced in tumors from PD-325901 treated animals or in the combination treated group compared to the control, but not significantly in the GDC-0941 treated group (Figure 7A). Concerning CC3, we could see an increase in PD-325901 and GDC-0941 treated mice, but the combination resulted the highest score even if that was not statistically significant (Figure 7B). We performed TUNEL staining to monitor DNA laddering, the main hallmark of apoptosis driven cellular death, and we could find a significantly elevated positive staining in tumors from the PD-325901 and the combination treated animals (Figure 7C).

**Figure 8:**
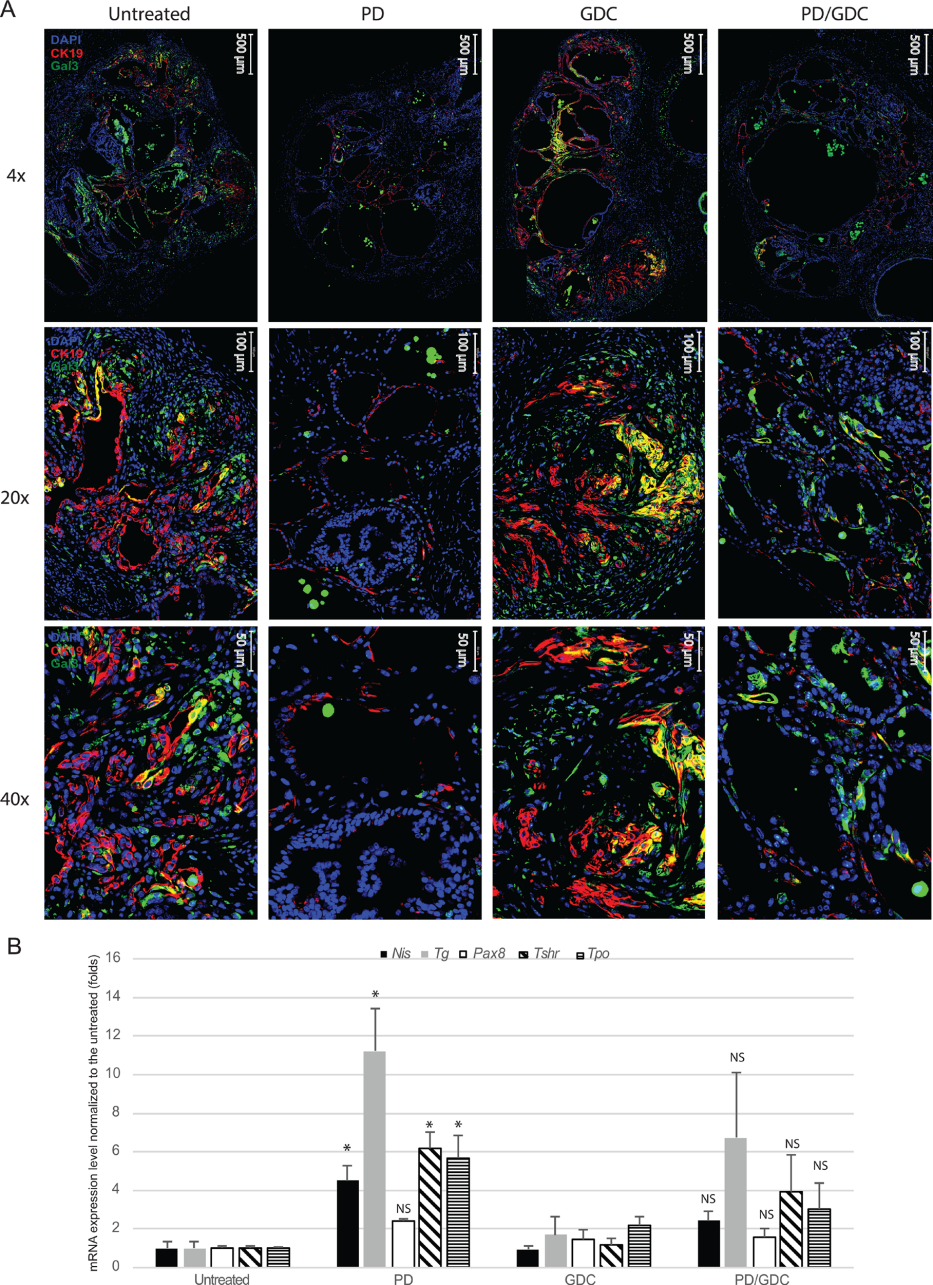
MEK inhibition alone or combined with Pi3′-kinase inhibition leads to a reduction in tumoral markers and an up-regulation of normal phenotype markers (**A**) Representative immunofluorescence images at 4×, 20× and 40× magnifications of isolated thyroid tissues from mouse treated 9 weeks with drugs. Blue: DAPI, Red: CK19, Green: Galectin-3. (**B**) mRNA expression levels of proteins involved in the normal function of the thyroid normalized to the untreated expressed in fold induction. Actin was used as house keeping gene and all measurements were done in triplicate and represented as the mean with SEM. One-way ANOVA test and Tukey test were used for multiple comparison.

To confirm the phenotype reversion found in PD-325901 treated thyroids, we performed staining for the tumor markers Cytokeratin 19 (CK19) and Galectin-3 (Gal-3), that are clinically used markers of thyroid cancer. While controls and GDC-0941 treated samples showed clear positive staining, PD-325901 and combination treated animals showed drastically reduced positivity for CK19 and very low positivity for Galectin-3 (Figure 8A). In fact, Galectin-3 positive cells observed in PD-325901 and combination treated samples are immune cells (e.g. macrophages) trapped in the cystic areas. This reversion was also tested by measuring thyroid specific markers by qPCR. PD-325901 induces the re-expression of all normal thyroid markers tested: Sodium Iodide Symporter (*Nis*), transcription factor Paired Box 8 (*Pax8*), Thyroid Stimulating Hormone Receptor (*Tsh-r*), Thyroid Peroxidase (*Tpo*) and Thyroglobulin (*Tg*). All of these genes, but *Pax8*, showed elevation of the transcription with MEK inhibition treatment, while GDC-0941 showed almost no alteration. Combination treatment showed elevations but not higher than MEK inhibition alone (Figure 8B).

## DISCUSSION

The management of ATC with multimodality treatment has so far failed to improve survival in patients. The lack of randomized studies prevents clinicians from being conclusive about the benefits of the available treatments including surgery. To improve the outcome, rationally targeted therapies are needed. Well-differentiated thyroid carcinomas are treated effectively by surgical resection and radioiodine therapy but there are limitations in using this strategy in ATC and radioiodine resistant PTC. The local invasiveness characterizing the undifferentiated type (ATC) is making it almost inoperable because of the presence of vital vessels and nerves. Understanding the key genetic events promoting the dedifferentiation and consequently progression and the maintenance of aggressive kinds of thyroid cancers is therefore crucial for the development of new effective treatments.

Currently, more and more efforts are focusing in the development of combination treatments targeting more than one target and/or pathway. This trend in cancer research is based on the fact that mono-chemotherapy is often associated with resistance, recurrence and sometimes worsening of the disease.

Synergy in combining anticancer drugs has to be uncovered empirically taking advantage of pre-clinical models like cell culture and genetically engineered mice. Previously published work has shown that BRAF^V600E^ and PIK3CA^H1047R^ mutations collaborate to promote rapid PTC formation and eventual ATC progression [20]. Moreover, these mutations are frequently found in ATC [28] with 25% for BRAF^V600E^ and 15% for PIK3CA^H1047R^. In addition, *PIK3CA* is also found frequently amplified in ATC [29]. Targeting both the RAF→MEK→ERK and the Pi3′-kinase→AKT→mTOR pathways is therefore a good rational to tackle aggressive forms of thyroid cancer.

The synergistic effect observed when targeting both pathways in two ATC cell lines demonstrates that the two pathways are needed to drive proliferation (Figure 1A–1B). Interestingly, synergy was stronger in OCUT-2 cell line, the only cell line harboring both the BRAF^V600E^ and the PIK3CA^H1047R^ mutations emphasizing the oncogene addiction effect. Nonetheless, the synergistic effect was observed in 8505c and an additive effect close to synergy was found in SW1736. As the later have no known mutations in PIK3CA, this suggest that these cell lines still require “wildtype” Pi3′-kinase activity to maintain proliferation. A similar synergistic effect was observed in thyroid cancer cell lines using RAF inhibitors (RAF265 and ZSTK474) with a Pi3′-kinase inhibitor (SB590885) in two PTC cell lines and in 8505c [30] or using a MEK inhibitor (AZD6244) with GDC-0941 on OCUT-1 cells and other PTC cell lines [31]. This shows that a variety of thyroid cancer cell lines are responding synergistically to drugs targeting the same (or similar) signaling nodes, thus supporting our presented data. Interestingly, it was also previously shown that blocking Pi3′-kinase can help overcome the resistance of some thyroid cancer cell lines to Tumor Necrosis Factor-Alpha-Related Apoptosis-Inducing Ligand (TRAIL) signaling (Lexatumumab), confirming the crucial role of Pi3′-kinase involvement in thyroid cancer cells’ drugs response [32].

Since cell cycle analysis could not explain the observed synergy: G1 arrest with marginal further effect when combined (Figure 1C–1D); we aimed at investigating drug-induced cell death mechanisms in the three ATC cell lines. To that end we evaluated cell death induction by performing annexinV/PI staining. An apoptotic effect of the drugs in the three cell lines could be seen 48h after treatment and, interestingly in these cases, the proportion of early or late apoptosis or necrosis was quite variable between the 3 cell lines, but ultimately the drug combination induced a superior effect, in terms of reducing the number of intact cells in the three cell lines than any drug alone (Figure 2B and Supplementary Figure 1).

In order to understand this drug-driven apoptosis induction, we investigated the effect of the treatments on cell signaling by western blotting. CC3 was moderately increased 24 h after treatment with MEK inhibitor but more when combined with the Pi3′-kinase inhibitor. Because the delay in apoptosis induction suggested that another mechanism was involved, we then looked for alternative cell death mechanisms. AIF does not involve caspases (its translocation into the nucleus leads to DNA fragmentation). However, none of our different treatments had any influence in both total and nuclear AIF levels suggesting no involvement of this mechanism. Gamma-H2AX, a marker of DNA damage which is a late event shared by all cell death mechanisms [33], increased with MEK inhibitor alone and further with the combination. We then investigated mechanisms involved in the regulation of CC3 activity. We found that Survivin, a member of inhibitor of apoptosis protein family (IAP) that sequestrates caspases and prevents the activation of the cascade responsible for apoptosis [34], was downregulated with drug treatments (Figure 3). This effect relying on protein turnover could explain the delayed apoptosis seen in cells (Figure 2).

This drug combination effect has never been tried in genetically engineered mouse model of thyroid cancer. We wanted to translate this synergistic effect observed in cells into a highly clinically relevant model *in vivo*. In a very similar fashion, both drugs showed a significant effect on tumor burden, but the combination showed an even more pronounced effect, that was statistically significant after 7 weeks of treatment (Figure 4A). Tumor growth was stabilized by GDC-0941 treatment, unlike the controls that kept on growing. This is a very interesting result showing that PIK3CA is not only important for the tumor in the initial phase [20] but that tumors still rely on its activity even a few months after induction. In addition, while untreated mice had several more aggressive regions looking like ATC at the end of the experiment (Figure 4C) GDC-0941 treated thyroids showed mainly PTC-like lesions. These observations also confirm the importance of the PIK3CA^H1047R^ mutation for ATC progression, as published previously [20]. The observed reduction in the tumor burden after PD-325901 treatment (Figure 4A), was due to a cell proliferation arrest (Figure 7A). This effect was potentiated by the combination with GDC-0941, with a more profound tumor burden reduction (60% for the combination versus 40% for PD-325901 alone) that could be explained by an apoptosis induction in addition to the PD-325901-dependent proliferation arrest (Figure 7A–7C), since the proliferation index was similarly affected. As shown by histology (Figure 4C), we were able to achieve a partial tumor remission in the time period considered (9 weeks) which is already a remarkable result considering the aggressive nature of the disease we were dealing with. Nevertheless, the remaining cells were still viable and resumed growth after drug removal (Figure 6). This implies that mutant cells could be tamed by MEK inhibition, but recover their aggressive behavior after drug removal. We think that MEK inhibition mainly pushes cells to re-differentiate. Then cells failing to repair their high genomic damage undergo apoptosis under the treatment pressure. Consequently, the high-grade tumor areas get killed and replaced by cells which succeeded to differentiate and/or the already resistant well differentiated tumor cells. The concomitant occurrence of re-differentiation and apoptosis was suggested by some studies however others propose that apoptosis is occurring later on [35, 36].

In terms of drug effect and pathways, we were able to show that MEK inhibition could maintain ERK1/2 phosphorylation levels to a practically undetectable level over the whole period of drug dosage (Figure 5A). We think that ERK1/2 inactivation promotes the re-differentiation of cells into functional thyrocytes. This is supported by the fact that thyroid cancer markers Cytokeratin-19 and Galectin-3 detection are strongly reduced upon MEK inhibition (Figure 6A). In addition, qPCR showed that normal thyroid markers that are usually lost in advanced thyroid cancers [37] are partially restored by the drug (Figure 8B), possibly rendering the tumor susceptible to radioactive iodine again. This is consistent with what has been previously published by the group of Prof. James Fagin in a PTC model [38]. Nevertheless, this is the first time that this effect is shown in a PTC/ATC mouse model where therapeutic options are extremely limited. In addition, we also show that while GDC-0941 induces a further decrease in tumor burden, it does not induce an additional beneficial effect in terms of thyroid marker expression, meaning that Pi3′-kinase does not play such a differentiation-driver role as ERK in the context of thyroid cancer. This was consistent with a recently published paper showing that complete ERK inhibition with a MEK inhibitor maximizes the responses of BRAF^V600E^ thyroid cancers to radioiodine by inducing sustained re-differentiation [39].

Unlike MEK inhibition, Pi3′-kinase inhibition could not maintain low AKT phosphorylation levels over time, meaning that another mechanism comes to compensate this. Still, the drug presents a strong effect in terms of tumor burden showing that Pi3′-kinase effect cannot be only reduced to AKT phosphorylation. A similar observation has been shown in another study by our group (Roelli *et al*, Manuscript in preparation). This uncoupling of Pi3′-kinase inhibition effect and AKT phosphorylation suggesting AKT independent actions of Pi3′-kinase would be a very interesting phenomenon to investigate in a future study.

In conclusion, we have demonstrated here that the combination of a MEK1/2 inhibitor with a PI3′-kinase inhibitor in the context of thyroid cancer is a valid approach that could relatively easy be translated into clinical practice. The advances in management of MEK inhibitors could allow such treatment to be realistically considered for patients, avoiding the toxic side effects (of current MEK inhibitors). The most interesting part is really the fact that MEK inhibition induces a kind of “re- diferentiation” of the thyroid, finally opening the door for new clinical managements of ATC cancer patients, a disease where the standard treatment of care has not changed much in the last 40 years.

## MATERIALS AND METHODS

### Chemicals

Unless specified otherwise, chemicals were purchased from Sigma-Aldrich Switzerland. MEK and Pi3′-kinase inhibitors, PD-325901 and GDC-0941 respectively, have been purchased from AbMole Bioscience Hong-Kong.

### Animals

Compound mice were obtained by combining the following alleles: *BrafCA* [40], *Pik3caLat* [41] and *Thyroglobulin-CreERT2* [42]. Mutations were induced by 5 consecutive daily injections of 1 mg tamoxifen IP. Mice were kept in isolated ventilated cages, fed ad libitum in a 12/12 h cycle of light and dark. Mice were kept, treated and euthanized according to the Swiss federal guidelines. The experimental protocol was approved by the Bernese cantonal ethical commission for animal experimentation (Licence number: BE120/13).

### Cell lines

8505c cells have a BRAF^V600E^ mutation in addition to p53 and CDKN2A alterations. Cells were purchased at the Public Health England repository (Culture Collections, Salisbury-UK) and cultured in RPMI 10% Fetal Bovine Serum (FBS), 2 mM L-glutamine, 1% Non-Essential Amino Acids (NEAA) and 1% Penicillin/Streptomycin (P/S). OCUT-2 carries concomitant BRAF^V600E^ and PIK3CA^H1047R^ mutations which was donated by Prof. James Fagin (Memorial Sloan Kettering Cancer Center) validated by Single-nucleotide polymorphism (SNP) and cultured in DMEM medium supplemented with 10% FBS, 2 mM L-glutamine, 1% NEAA and 1% P/S. SW1736 is a BRAF^V600E^/PIK3CA^WT^ cell line purchased at the Cell Line Service (CLS, Eppelheim-DE) and cultured in RPMI 10% FBS, 2 mM L-glutamine and 1% P/S. All cell lines were cultured for a maximum of 40 passages or 6 months; whichever limit was reached first.

### Synergy calculation method

Five-fold serial working dilutions were prepared in RPMI-1640 medium. PD-325901 and GDC-0941 were used respectively at the following concentrations (100 nM, 20 nM, 4 nM, 0.8 nM, 0.16 nM) and (2 μM, 400 nM, 80 nM, 16 nM, 3.2 nM) in SW1736 and OCUT-2 cell lines. In 8505c the final concentrations used were (200 nM, 40 nM, 8 nM, 1.6 nM, 0.32 nM) for PD-325901 and (3 μM, 600 nM, 120 nM, 24 nM, 4.8 nM) for GDC-0941. These concentrations represent approximately (10, 2, 0.4, 0.08 and 0.016) times fold of the calculated IG50 for each drug in each cell line. Cells were incubated 72 h then formalin-fixed and stained with crystal violet. Crystal violet was recovered in Sodium Dodecyl Sulphate 1%. Optical density at 550 nm was measured after lysis and normalized to the untreated cells. Curves of percentage of inhibition as a function of IG50 equivalents were obtained. This constant ratio approach allows to calculate the combination index (CI) values. CI = (D)_1_/(D_x_)_1_ + (D)_2_/(D_x_)_2_ [26].

At 50% inhibition level, (D_x_)_1_ and (D_x_)_2_ are the concentrations of PD-325901 and GDC-0941, respectively that induce alone a 50% inhibition of cell growth; (D)_1_ and (D)_2_ are concentrations of PD-325901 and GDC-0941 in combination, which also inhibits cell growth by 50% (D)_1_/(D_x_)_1_ reflect how much Drug 2 potentiate the affect of Drug 1 and (D)_2_/(D_x_)_2_ reflect how much Drug 1 potentiate Drug 2 in combination. When the 2 drugs potentiate each other (Synergy) each one lowers the concentration of the other which is necessary to reproduce the same effect as a single agent.

The combination index was calculated for each percentage of inhibition or fraction affected (Fa). Synergism or antagonism was calculated on the basis of the multiple drug-effect equation and evaluated by the CI, where CI < 1, CI = 1, and CI > 1 indicate respectively synergism, additivity and antagonism using Compusyn 2.0 software based on the Chou and Talalay method.

### Cell cycle analysis by propidium iodide (PI) staining

After 24 h, the medium was centrifuged for 5′ at 300 g to recover the floating cells. Attached cells were trypsinized (Trypsin 0.05%) and recovered with medium. After 5′ of centrifugation at 300 g, the pellets were re-suspended in PBS, and counted using a Neubauer chamber. Then attached cells were pooled with the floating cells and centrifuged for 5′ at 300 g. Cells were then re-suspended in cold PBS at 1 million cells/ml. Then, cells were centrifuged for 5′ at 300 g and the pellet was re-suspended in 0.3 ml cold PBS /million cells. Then 0.7 ml cold ethanol 70% per million cells was added drop wise while vortexing gently. The suspensions were aliquoted in 1 ml aliquots and left over night at 4°C.

The tubes were centrifuged 10′ at 1,000 g at 4°C and the supernatants were decanted. Pellets were washed once with 1 ml cold PBS 1x and then centrifuged 10′ at 1,000 g at 4°C. Cells pellets were re-suspended in 250 μl of cold PBS 1x and 5 μl of 10 mg/ml RNase A was added. After an incubation of 30′ at 37°C, 10 μl of PI solution (40 μg/ ml) was added, the cells were then incubated 10′ at room temperature in the dark and then analyzed using FACS (BD FACScan equipped with a Cytek solid state laser, and FCS Express De Novo software).

### AnnexinV/Propidium iodide staining for flow cytometry analysis

Cells were treated with PD-325901 and/or GDC-0941 at 100 nM and 1 μM concentration respectively for 24 h or 48 h. Floating cells were centrifuged at 1,000 g for 10′. Attached cells were washed with PBS and trypsinized before re-suspending them in the appropriate media. Cells suspensions were centrifuged at 300 g for 10′, re-suspended in cold PBS before a second round of centrifugation at 300 g for 10′. The fractions of floating cells and attached cells were pooled in 1X annexinV binding buffer and re-suspended. Cells were counted and 100 μl of cell suspension containing 10^5^ cells was transferred to a 5 ml culture tubes. BD Pharmingen FITC annexinV Apoptosis Detection Kit I (BD Biosciences) was used for the staining. 5 μl of FITC annexinV and 10 μl of PI were added to the suspension, gently vortexed and incubated for 15′ at room temperature in the dark before measuring by flow cytometry within 1 h.

### Total protein preparation from cells

After 24 h of treatment, both floating and attached cells were recovered. They were then centrifuged for 5′ at 350 g at 4°C and washed twice with 10 ml cold PBS and re-suspended in 1 ml cold PBS. Cells were then pelleted at 500 g and lysed in RIPA buffer with Halt™ Protease and Phosphatase Inhibitor Cocktail (Pierce, ThermoFischer Scientific). The lysates were incubated 30′ on ice and then centrifuged for 15′ at 16,000 g at 4°C. Supernatants were recovered, and protein concentrations were quantified by the Bradford method (Pierce, ThermoFischer Scientific, BCA Protein Assay Kit).

Lysates were prepared at 1-4 μg/μl in sample buffer (5x; 1.47 M sucrose, 10% SDS, 5 mM EDTA, 300 mM Tris pH 8.8, 0.25% Bromophenol blue, and 130 mM dithiothreitol) and the proteins were run on TGX precast 4-20% gels (BioRad, Switzerland), transferred onto Trans-Blot transfer pack nitrocellulose (BioRad, Switzerland), and analyzed by western blotting.

### Nuclear extract preparation

After 24 h of treatment, cells were washed twice with cold PBS 1x. Then they were scrapped in PBS and centrifuged for 5′ at 350 g at 4°C. The pellets were re-suspended in 500 μl of hypotonic buffer (20 mM Tris pH 7.4, 10 mM NaCl, 3 mM MgCl_2_) and incubated for 15′ on ice. Then, 25 μl of 10% IGEPAL^®^ CA-630 was added to each tube and after vortexing for 10 s at highest speed, they were centrifuged for 10′ at 1,000 g at 4°C. The pellets were re-suspended in 40-80 μl of RIPA/Halt™ (Halt cocktail, ThermoFischer Scientific). Finally, they were cleared at 16,000 g at 4°C for 30′. The protein concentrations and the preparation of the lysates were performed as described above.

### Drug treatments and ultrasound imaging

Mice were treated by oral gavage with 5 mg/kg of PD-325901 or 50 mg/kg of GDC-0941, or the combination of both, formulated in 5% hydroxpropyl methylcellulose 6 days per week. Mice were anesthetized with 5 μl/g body weight of a mixture of 0.1 mg/ml Dorbene, 0.5 mg/ml Dormicum and 5 μg/ml Fentanyl in 0.9% NaCl injected intraperitoneally. The fur around the neck was epilated with Veet^®^ hair removal cream. Pictures were acquired with an ESAOTE MyLab Five ultrasound machine using a LA455 Probe (18 MHz) from Siemens. After imaging, mice were taken out of anesthesia with 10 μl/g body weight of a mixture of 0.25 mg/ml Alzan, 5 μg/ml Flumazenil and 20 μg/ml Naloxon in 0.9% NaCl injected sub-cutaneously. Images were analyzed using the ImageJ software. Tumor burden was assayed as the surface of the biggest thyroid section observed and normalized to the size at the beginning of the experiment.

### Immunofluorescence

Mice were anesthetized using 10 μl/g of bodyweight of a Ketamin/Xylazin mixture (10 mg/ml and 1.6 mg/ ml respectively) injected intraperitoneally. Thyroids were dissected and washed with ice cold PBS. Tissues were fixed overnight in 10% neutral buffered formalin solution (Sigma). After paraffin embedding, 5 μm sections were prepared. Sections were rehydrated and targets were retrieved in TEG buffer (10 mM Tris and 0.5 mM EGTA). Sections were blocked three times 10′ in a buffer of 1% BSA, 0.2% gelatin, and 0.05% Saponin in PBS. Antibodies were diluted in 0.1% BSA and 0.3% Triton X-100 in PBS to the following concentrations: KI67 (Abcam-16667 1:300), CC3 (CST-9664S 1:300), CK19 (TROMA-III DSHB 1:300), Galectin-3 (Abcam-53082 1:300). Primary antibodies were incubated over night at 4°C. The slides were washed three times with 0.1% BSA, 0.2% gelatin and 0.05% Saponin in PBS. The secondary antibody was goat anti Rabbit 488 Life Technologies A-11034 1:500. DAPI Sigma 32670 was used at a final concentration of 5 μg/μl. Secondary antibodies were incubated 1 h at room temperature.

### Microscopy

Immunofluorescence and histology pictures were scanned with a Panoramic Midi Scanner (Sysmex/3DHISTECH Switzerland/Hungary). Analysis and quantification was performed on whole tumor sections with the QuantCenter software (Sysmex/3DHISTECH Switzerland/Hungary).

### Hematoxylin-Eosin Staining

For histological analysis, tissue samples were processed as described above and stained with Hematoxylin (Sigma GHS132) and Eosin (HT110132) following standard protocols.

### Antibodies for western blots

Primary antibodies (dilution and catalogue number; all CST antibodies were purchased from Bioconcept AG, Allschwil, Switzerland): p44/42 MAPK (Erk1/2) (1:5000; CST-9107), phospho-p44/42 MAPK (Erk1/2) (1:2000; CST-4370), AKT (pan) (1:2000; CST-2920), phospho-AKT (Ser473) (1:2000; CST-4060), Phospho-4E-BP1 (Thr37/46) (1:2000; CST-2855), phospho-S6 (Ser240/244) (1:6000; CST-4858), XIAP (1:1000, CST-2042), CC3 (1:2000; CST-9664), gamma H2A.X (Phospho S139) (1:1000; Abcam-26350), AIF (1:2000, Abcam-32516), Survivin (1:500; Santa Cruz 17779).

Secondary antibodies were from Li-Cor Biosciences, Bad Homburg Germany: IRDye 680RD Goat anti-Rabbit IgG (H + L) (1:10,000, 926-68071), IRDye 800CW Goat anti-Mouse IgG (H + L) (1:10,000, 926-32210).

### Real time PCR

Total RNAs were purified with the QIAzol reagent from Qiagen according to the manufacturer's instructions. For RT-PCR experiments, total RNA (500 ng) was subjected to reverse transcription to produce cDNA using Oligo(dT)12-18 Primer and the Super Script II Reverse Transcriptase (Invitrogen^®^) then run on ViiA™ 7 Real-Time PCR System (Applied Biosystems, Life Technologies) using the TaqMan^®^ gene expression Master Mix and primers from Applied Biosystems. Actin was used as housekeeping gene and mean C_T_ Values and standard deviations are used in the ΔΔCT calculation (comparative C_T_ method). Each sample was run in triplicate. The primers used were purchased from Applied Biosystems, Life Technologies: *Nis* (Mm01351811_m1), *Tg* (Mm01200340_m1), *Ttf1* (Mm00657018_m1), *Pax8* (Mm00440623_m1), *Tpo* (Mm00456355_m1), *Tshr* (Mm00442027_m1), and *Actb* (4352341E).

### Statistical methods

Two-way ANOVA test with Tukey as post hoc test were used to compare tumor burdens between the 4 treatments at for different time points (Figure 4). One-way ANOVA test with Dunnett as post hoc test was used for the quantification of pERK and pAKT western blots at 9 weeks (Figure 5) and for the FACS analysis apoptosis (Figure 2). Otherwise, a Student's ttest was used to compare pERK and pAKT in thyroids of mice treated with a single dose GDC (Figure 5). One-way ANOVA test and Tukey test were used in all other statistical analysis (Cell cycle Figure 1; Immuno stainings Figure 7; qPCR Figure 8).

All statistical analyses were performed using the software Graphpad Prism. Significance in the figures is displayed using stars with one star meaning a resulting *p-value* of smaller than or equal to 0.05. Two stars means a *p-value* of smaller than or equal to 0.01. Three stars stands for a *p-value* of smaller than or equal to 0.001. If a *p-value* was greater than 0.05 the difference between the two concerning groups was regarded as not significant.

## SUPPLEMENTARY MATERIALS FIGURES


