# Supplementary material for: Combined MEK and Pi3′-kinase inhibition reveals synergy in targeting thyroid cancer in vitro and in vivo
Source: Oncotarget. 2017 Feb 21;8(15):24604–20. doi: 10.18632/oncotarget.15599 (PMC5421873; doi:10.18632/oncotarget.15599)
Supplement: Supplementary file 1 [file oncotarget-08-24604-s001.pdf]

Combined MEK and Pi3'-kinase inhibition reveals synergy in targeting thyroid cancer *in vitro* and *in vivo*

Supplementary Materials

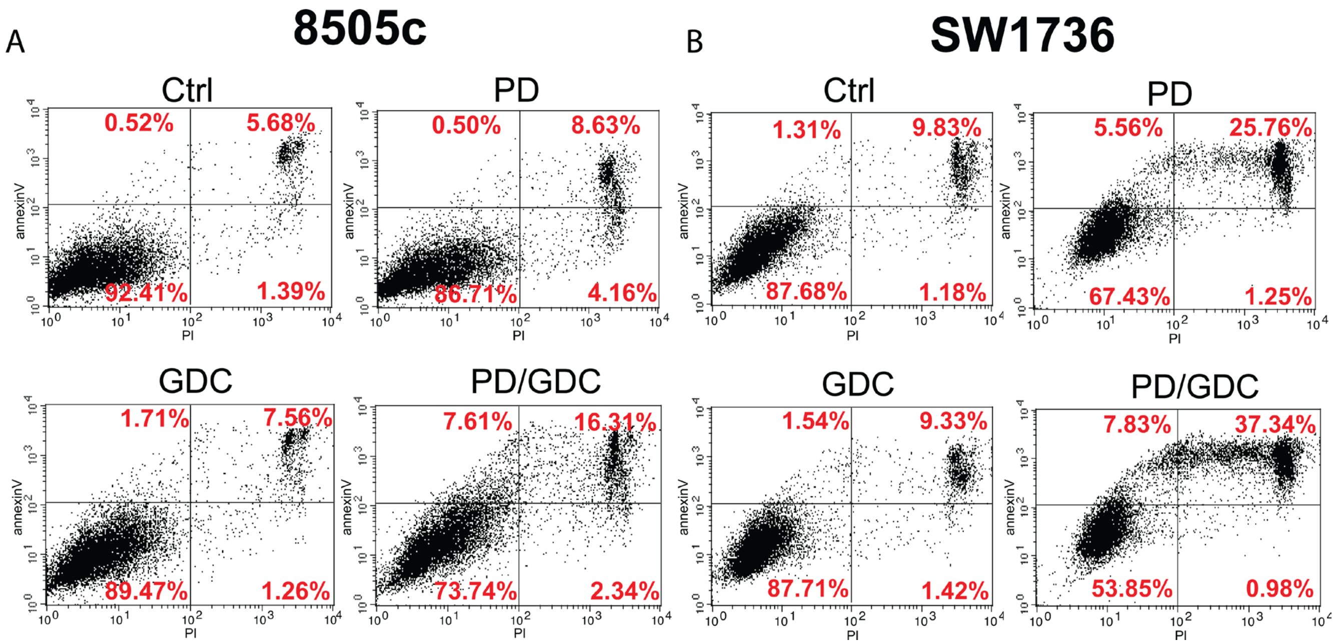

Supplementary Figure 1: Drug combination synergistically induces apoptosis only after 48 h in 8505c and SW1736 anaplastic thyroid cancer cell lines. (**A–B**) Flow cytometry analysis of 8505c and Sw1736 cell lines treated with 100 nM of PD-325901, 1 µM of GDC-0941 or the combination for 48 h stained with FITC annexinV and Propidium iodine.
